# Early activation of wheat polyamine biosynthesis during Fusarium head blight implicates putrescine as an inducer of trichothecene mycotoxin production

**DOI:** 10.1186/1471-2229-10-289

**Published:** 2010-12-30

**Authors:** Donald M Gardiner, Kemal Kazan, Sebastien Praud, Francois J Torney, Anca Rusu, John M Manners

**Affiliations:** 1 CSIRO Plant Industry, Queensland Bioscience Precinct, 306 Carmody Road, St. Lucia, Brisbane, 4067, Australia; 2 Biogemma, Site ULICE, ZAC les portes de Riom-BP173, 63204 Riom, France

## Abstract

**Background:**

The fungal pathogen *Fusarium graminearum *causes Fusarium Head Blight (FHB) disease on wheat which can lead to trichothecene mycotoxin (*e.g*. deoxynivalenol, DON) contamination of grain, harmful to mammalian health. DON is produced at low levels under standard culture conditions when compared to plant infection but specific polyamines (*e.g*. putrescine and agmatine) and amino acids (*e.g*. arginine and ornithine) are potent inducers of DON by *F. graminearum *in axenic culture. Currently, host factors that promote mycotoxin synthesis during FHB are unknown, but plant derived polyamines could contribute to DON induction in infected heads. However, the temporal and spatial accumulation of polyamines and amino acids in relation to that of DON has not been studied.

**Results:**

Following inoculation of susceptible wheat heads by *F. graminearum*, DON accumulation was detected at two days after inoculation. The accumulation of putrescine was detected as early as one day following inoculation while arginine and cadaverine were also produced at three and four days post-inoculation. Transcripts of ornithine decarboxylase (ODC) and arginine decarboxylase (ADC), two key biosynthetic enzymes for putrescine biosynthesis, were also strongly induced in heads at two days after inoculation. These results indicated that elicitation of the polyamine biosynthetic pathway is an early response to FHB. Transcripts for genes encoding enzymes acting upstream in the polyamine biosynthetic pathway as well as those of ODC and ADC, and putrescine levels were also induced in the rachis, a flower organ supporting DON production and an important route for pathogen colonisation during FHB. A survey of 24 wheat genotypes with varying responses to FHB showed putrescine induction is a general response to inoculation and no correlation was observed between the accumulation of putrescine and infection or DON accumulation.

**Conclusions:**

The activation of the polyamine biosynthetic pathway and putrescine in infected heads prior to detectable DON accumulation is consistent with a model where the pathogen exploits the generic host stress response of polyamine synthesis as a cue for production of trichothecene mycotoxins during FHB disease. However, it is likely that this mechanism is complicated by other factors contributing to resistance and susceptibility in diverse wheat genetic backgrounds.

## Background

*Fusarium *head blight (FHB) or scab is one of the most important diseases of wheat and other small grain cereals in many wheat growing regions [1]. FHB is caused mainly by *F. graminearum*, but also by other related *Fusaria*. A characteristic of FHB disease is the production of trichothecene mycotoxins such as deoxynivalenol (DON) by the fungus in infected heads. Trichothecenes are phytotoxic [2] and their biosynthesis during FHB is necessary for full pathogen virulence, and the spread of the fungus through the infected wheat head [3-5]. Importantly, trichothecenes such as DON are toxic to animals and humans when present in feed and food products, respectively [6]. Because of the undesirable effects of DON on human and animal health, its presence in grain is tightly regulated in many wheat grain markets [7]. Consequently, there is also a strong interest in the development of novel technologies for reducing DON levels in infected wheat either through breeding or via chemical and/or biological control methods [8,9].

**Figure 1:**
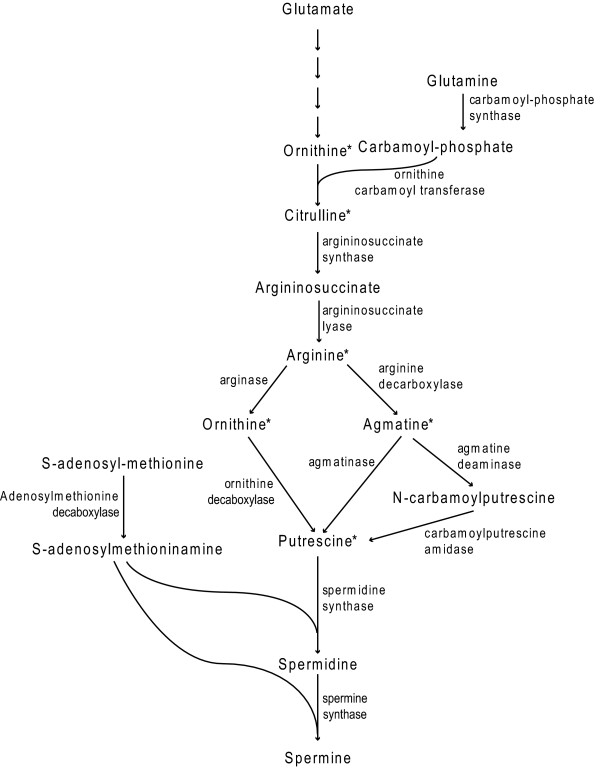
**Principle pathway of polyamine biosynthesis in plants**. Expression of all genes encoding enzymes of the pathway were investigated except for agmatine deaminase, carbamoylputrescine amidase and spermine synthase. Metabolites measured in this study included arginine, putrescine, spermine and spermidine. Metabolites indicated by an asterisk were previously shown to have *in vitro *trichothecene inducing activity [18].

One constraint on our ability to reduce DON production by *Fusarium *pathogens has been a limited understanding of environmental and host-associated genetic factors that regulate the production of trichothecene toxins during the infection process [10]. It is well known that the amount of DON produced by *F. graminearum *during the infection of living wheat plants is much higher than that observed under common culture conditions, including growth on autoclaved wheat grains. This suggests that specific host factors stimulate DON production during the infection process [11,12]. Several factors that may induce the production of DON by *F. graminearum *during the infection process have been proposed. These factors include hydrogen peroxide [13], sugars [14], acidic pH [15,16], and fungicides [17]. In particular, we have recently shown in a screen of various nitrogen containing compounds that the most potent inducers of DON production by *F. graminearum *were metabolites (*e.g*. arginine, ornithine, agmatine, citrulline and putrescine) of the plant polyamine biosynthetic pathway shown in Figure 1[18]. These metabolites of the polyamine pathway appear far more potent than hydrogen peroxide and sugars at inducing DON production *in vitro *[18]. Importantly, the levels of DON produced in culture filtrates of *F. graminearum *after growth in the presence of inducing amines such as agmatine, putrescine and ornithine were extremely high (> 500 ppm) [18]. The primary biosynthetic enzyme in trichothecene biosynthesis in *F. graminearum *is trichodiene synthase [19] encoded by *TRI5 *[5] and transcripts of *TRI5 *were also induced in the fungus by polyamines in culture to levels equivalent to those observed in infected heads [18]. Interestingly, both spermine and spermidine, two very common and abundant plant polyamines, did not induce DON production, suggesting that not all polyamines were inducers of DON [18]. Nevertheless, the stimulation of trichothecene biosynthesis by specific polyamines appears to be a general response in *Fusarium *pathogens because the DON-inducing polyamine agmatine was also able to induce the production of T-2 toxin, another trichothecene, in *F. sporotrichioides *[18]. Because of the potency of polyamine inducers in stimulating trichothecene mycotoxins in culture, Gardiner et al. [18] hypothesised that the pathogen may perceive polyamines and related amino acids as cues for the production of toxins during the infection process.

Polyamines are well known as metabolites rapidly induced by diverse abiotic stresses in plants, including salinity, drought, chilling, hypoxia, ozone, heavy metals and UV irradiation [20,21]. The biosynthetic pathway of the principle polyamines of plants is well understood (Figure 1). Two routes of synthesis to the primary amine putrescine have been described with the first steps being decarboxylation of either ornithine or arginine catalysed by ornithine decarboxylase (ODC) and arginine decarboxylase (ADC), respectively. In subsequent reactions aminopropyl groups are generated from *S*-adenosylmethionine (SAM) by SAM decarboxylase to convert putrescine to spermidine and subsequently spermine. Cadaverine is thought to be produced by the decarboxylation of lysine catalysed by lysine decarboxylase in prokaryotes but in eukaryotes this step is less well defined [22,23]. A role for polyamines in protection against the stress-induced cellular damage has been demonstrated in transgenic plants of rice, Arabidopsis, tobacco, tomato and pears that accumulate high levels of polyamines through the over-expression of key biosynthetic enzymes in the polyamine biosynthetic pathway [reviewed by [24]].

In contrast to abiotic stresses where polyamine accumulation is a general stress response, the response of polyamines to pathogen challenge appears to be more dependent on the host-pathogen system under study. Increases in polyamine content in susceptible barley during rust and powdery mildew disease development [reviewed by [25]] and in rice after infection with the blast pathogen *Magnaporthe grisea *[26,27] have been reported. In contrast, infection of tobacco with powdery mildew and downy mildew pathogens as well as the necrotroph *Alternaria tenuis *led to decreased levels of polyamines [28]. Increasing polyamine levels via over-expression of polyamine biosynthetic enzymes has been shown to increase the tolerance of tobacco to *F. oxysporum *[29]. Conjugates of agmatine, such as hordatine from barley and feruloylagmatine from wheat, are also known to have antifungal activity [30,31]. Furthermore, polyamine biosynthesis appears to be positively regulated by the plant defence hormone methyl jasmonate in wheat and barley but not in rice [32-34]. Polyamine oxidases are also thought to contribute to reactive oxygen species generation during defence against diverse pathogens [26].

Because polyamines are generically induced during plant stress, it is possible that *Fusarium *pathogens have evolved to recognise these metabolites during the infection of wheat plants to trigger toxin production. However, it is currently unknown what polyamines or related metabolites accumulate during FHB development. Gardiner et al [18] described a preliminary experiment that suggested that putrescine was elevated in wheat heads during FHB disease development, but only one metabolite and one post-inoculation time-point was analysed. In the present study, we have studied the expression of wheat genes that encode polyamine biosynthetic enzymes during FHB development to determine if this host pathway is activated during infection. We have also analysed a spectrum of polyamines and amino acids at multiple time-points after inoculation and across multiple bread wheat genotypes. These experiments have permitted a comparison of the temporal and quantitative patterns of accumulation of polyamines and amino acids during infection with the timing and concentrations of DON in infected heads. The results demonstrate that the core polyamine biosynthetic pathway is activated early on during *F. graminearum *infection in wheat and that putrescine accumulation occurs prior to toxin production by the pathogen. This latter observation provides additional support to the view that polyamines are not only inducers of toxin production by the pathogen *in vitro *but may also play a similar role *in planta*.

## Results

### Polyamine, amino acid and DON accumulation during FHB disease development

The observation that intermediates of the polyamine pathway are strong inducers of *TRI5 *expression and DON production by *F. graminearum *in culture led us to investigate the concentrations of these compounds in wheat heads during infection. Inflorescences of a susceptible wheat cultivar were spray inoculated with conidia of *F. graminearum *at mid-anthesis and polyamines and free amino acids were analysed in head samples taken daily over a seven-day period.

**Figure 2:**
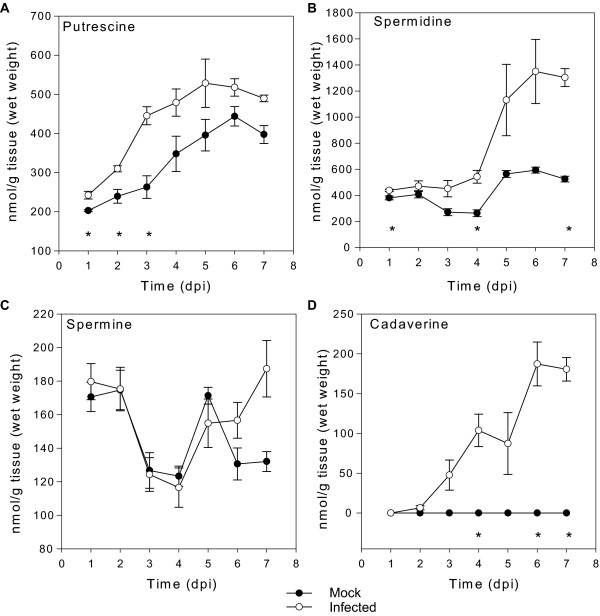
**Quantification of polyamines in wheat heads during Fusarium head blight disease development**. Asterisks indicate significant differences (t-test *P *< 0.05). Error bars are the standard error of the mean. n = 4 individual heads.

The putrescine concentration in the infected spikes increased rapidly and was almost two fold that of the mock inoculated control at three days post-inoculation and then reached a plateau at approximately 500 nmoles/g fresh weight (Figure 2A). Concentrations of spermidine increased more slowly and appeared to be higher than mock inoculated controls after four to seven days post-inoculation. Spermidine was the most abundant polyamine reaching a level of 1400 nmoles/g fresh weight. Spermine levels were more variable and no significant differences were observed in its concentration in infected relative to mock-inoculated heads (Figure 2C). The levels of putrescine and spermidine increased during the seven-day period even in the mock inoculated heads, indicating that polyamine accumulation may be a normal part of development (Figure 2A-B). Similar increases in these polyamines were observed during the early stages of grain development in field grown wheat [35] and also during development of the rice panicle [36]. Putrescine is an inducer of DON *in vitro *and it was interesting that inoculation led to a rapid increase in putrescine levels which were significantly higher than those of mock-inoculated controls at one, two and three days post-inoculation (Figure 2A). Agmatine is also a potent DON inducer and a precursor of putrescine synthesis but using our extraction methods we were unable to detect agmatine in wheat heads (data not shown). This is most likely due to the instability of agmatine, as observed by others as well [37]. Interestingly, another polyamine and potent inducer of DON production, cadaverine [15], was not detected in the mock-inoculated heads at all but accumulated in infected heads, with significant increases observed at three days post-inoculation, eventually reaching a concentration of 200 nmoles/g fresh weight. Quantitative RT-PCR measurement of fungal polyamine biosynthetic gene expression indicated a lack of induction of fungal transcripts relative to those of the host during infection (data not shown) and coupled with plant material being the dominant contributor to biomass at all time points, this suggests that the measured polyamines are most likely almost exclusively of plant origin.

**Figure 3:**
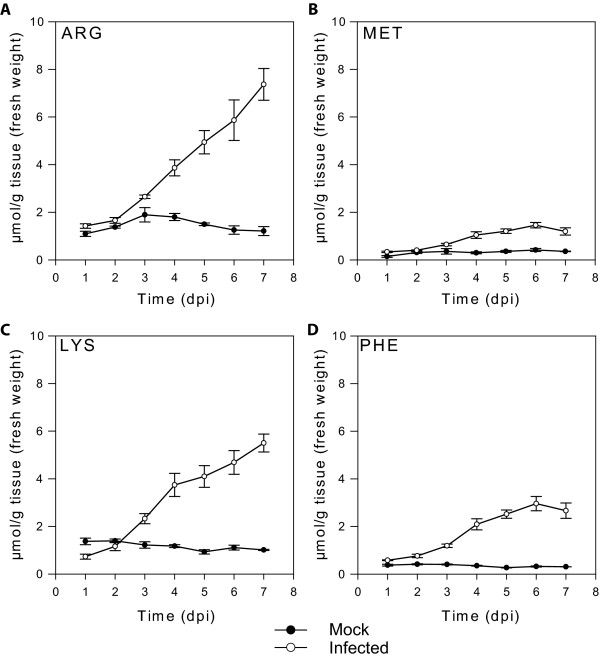
**Free amino acids during Fusarium head blight of wheat**. Error bars are the standard error of the mean. n = 4 individual heads.

We also analysed the amino acid content of these heads because arginine was a strong DON inducer while some other amino acids such as lysine, methionine and phenylalanine were weak DON inducers [18]. In addition, combinations of amino acids and polyamines (e.g. methionine with putrescine) appeared to act synergistically for DON induction in culture [18]. Unlike polyamines, most amino acids did not show any increase in concentration during the development of the head in mock-inoculated controls. Some amino acids did increase in concentration during FHB disease development and these included glycine, valine, arginine, alanine, phenylalanine, lysine and leucine as well as threonine and/or citrulline which could not be resolved (Figure 3 and Additional File 1). However, a significant increase in the concentration of most of these compounds, and particularly the potent DON inducer arginine, was only observed later in the infection time-course (Figure 3). The concentration of arginine increased from approximately 1 μmol/g fresh weight at one day post-inoculation to up to 7 μmol/g fresh weight at seven day post-inoculation. The only amino acid that rapidly responded to inoculation was isoleucine where an increase was observed at one day post-inoculation but this was not sustained at later stages of infection (Additional File 1). Ornithine is another potent DON inducer [18] but this amino acid was below the detection level in wheat heads using our instrumentation. Using more sensitive equipment (Waters AccQ-Tag Ultra at the Australian Proteome Analysis Facility), we were able to detect trace amounts of ornithine (maximum observed 0.05 μmol/g fresh wt).

**Figure 4:**
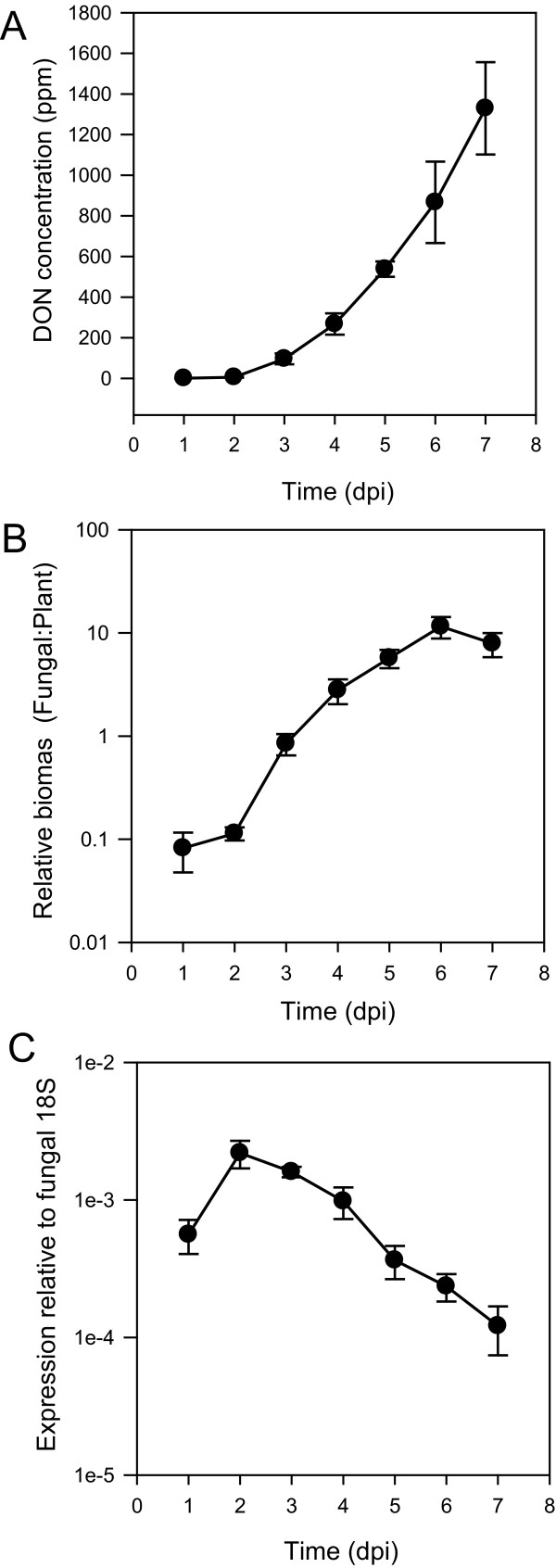
**DON concentration (A), fungal biomass (B) and *TRI5 *expression (C) during Fusarium head blight of wheat**. Error bars are the standard error of the mean. n = 4 individual heads.

In order to temporally compare the accumulation of potential DON inducing polyamines and amino acids measured in the time-course experiment (Figure 2 and Figure 3) with disease and DON levels, we also measured DON levels and fungal biomass (Figure 4) during disease development. DON was not detectable in the infected heads until three days post-inoculation and then steadily increased, reaching 1200 ppm in fresh head tissue at seven days post-inoculation. Fungal biomass in infected tissue was measured by quantitative PCR of a fungal DNA sequence relative to that of a plant sequence in DNA extracted from infected heads. Similarly, only minor increases in fungal biomass for the first two days post-inoculation was detectable but this increased rapidly thereafter and peaked at five to six days after inoculation. Therefore, these experiments suggest that the induction of putrescine in infected heads preceded the production of DON in the fungus.

### Induction of genes encoding polyamine biosynthetic enzymes during FHB

The biosynthesis of putrescine, spermine and spermidine from primary amino acid metabolism involves several enzymatic steps, with two potential branches of putrescine synthesis from arginine via either ornithine or agmatine as intermediates (Figure 1). We were interested in knowing which genes of this pathway might be activated by fungal infection and the timing of this response. To determine this, we first identified wheat homologues for 11 of the polyamine biosynthetic enzymes (Figure 1 & Table 1). We used previously annotated rice polyamine sequences as queries in searches of wheat expressed sequence tag clusters available in the WhETS database. Although not a definitive analysis of copy number, based on the clusters produced by WhETS for the query sequences all polyamine pathway genes described in this manuscript appear to be present in single copies in each of the three wheat homoeologous genomes. The wheat sequences identified in these analyses were used to design primers (Table 1) for Quantitative Real-Time Reverse Transcriptase PCR analysis of transcripts in RNA samples from mock- and *F. graminearum*-inoculated heads of the susceptible wheat cultivar Kennedy during FHB disease development.

**Table 1:** Quantitative reverse transcriptase-PCR primers used for detecting expressions from wheat polyamine biosynthesis genes.

Gene	Forward primer (5'-3')	Reverse primer (5'-3')	Locus	GenBank accession
Ornithine decarboxylase (ODC)	AGCGTTACTTCGGGGAGCTT	ATTGTGAAGGCGGTCTCG	Os09g37120	HM770451

Arginine decarboxylase 1 (ADC1)	CACCAAGATACCAGGCCACT	GTGGAAGTGCAGCAACTTGA	Os04g01690	HM770446

Arginine decarboxylase 2 (ADC2)	AGGAGGAGGAGCTCGACATT	GCCGAACTTGCCCTTCTC	Os06g04070	HM770449

Agmatinase/Arginase	GGGAAGAGATTTGGTGTGGA	TCACACCTTCCCCAAGTTTC	Os04g01590	HM770450

S-adenosylmethionine decarboxylase 2	GCGTCCTCATCTACCAGAGC	CTTGCCTTCCTTGACCAGAG	Os04g42090	HM770448

Spermidine synthase 1	TGATTCAGGACATGCTTTCG	CCCAATTGCACCACTAGGAT	Os02g15550	HM770442

Spermidine synthase 2	GCAAAAATCCAATGACACCA	TGTGGCTGCACACACATCTA	Os06g33710	HM770452

Argininosuccinate synthase	ACGCTGAAGGTTTCATCAGG	TAGATGCCCTTCTCCAGCAT	Os12g13320	HM770445

Argininosuccinate lyase	GTTGAACAGTTGGAGCGTGA	GAGTCCAGTTCCAGCCAAAG	Os03g19280	HM770447

Carbamoyl-phosphate synthase	TTGGAAAATTGTTGGTGTTG	CCCATTCATATGGAGCATC	Os02g47850	HM770444

Ornithine carbamoyl transferase	ATGAAGCCCTGATGGAGATG	ATGGCACCGTCTGTTACCTC	Os02g47590	HM770443

18 S rRNA*	CGACCTACTCGACCCTTCGGCCGG	CGATGCCGGAAACACGACCCGG	-	

Peroxidase† (TaPERO)	GAGATTCCACAGATGCAAACGAG	GGAGGCCCTTGTTTCTGAATG	-	X56011

18 S rRNA fungal*	GTCCGGCCGGGCCTTTCC	AAGTCCTGTTTCCCCGCCACGC	-	

TRI5*	CACTTTGCTCAGCCTCGCC	CGATTGTTTGGAGGGAAGCC	FGSG_03537	

* Primers from Mudge et al. [11]. †Primers from Pritsch et al. [42]

**Figure 5:**
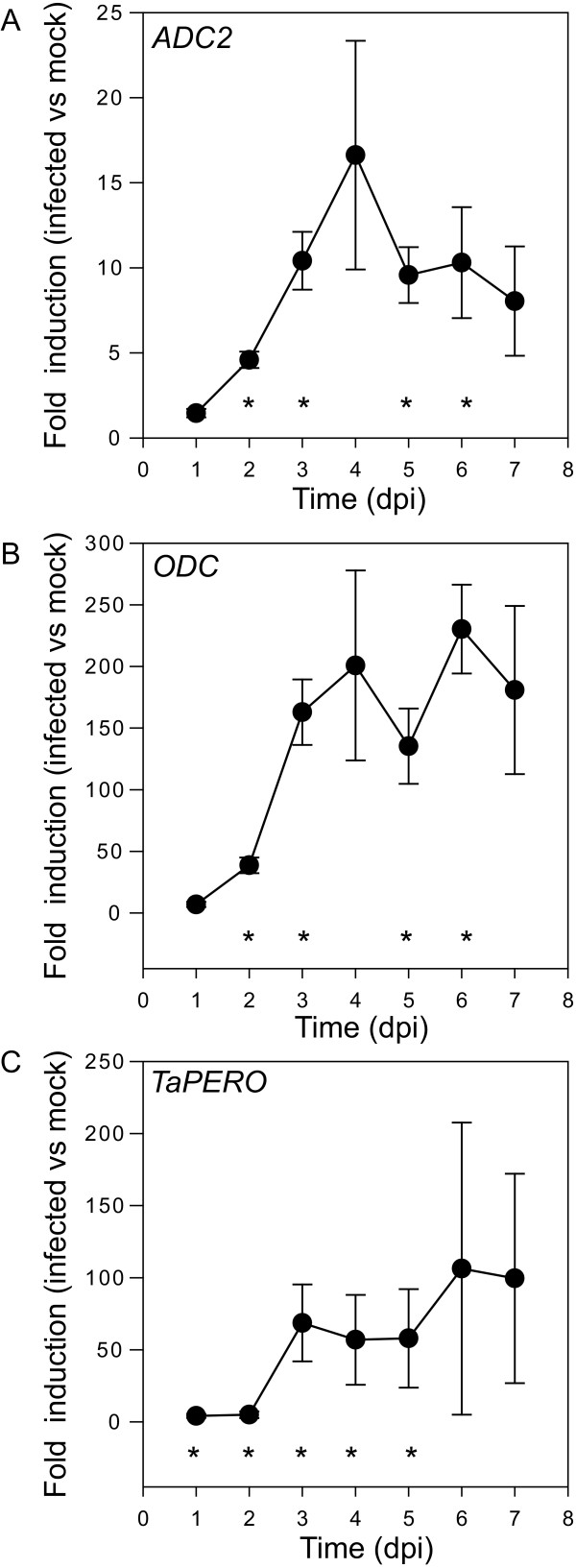
**Expression of genes of the polyamine biosynthesis pathway during Fusarium head blight infection**. Values are the relative expression between infected plants compared to mock inoculated plants. Error bars are the standard error of the mean. n = 4 individual heads. Asterisks indicate statistically significant (t-test *P *< 0.05) differences between infected and mock treated samples at that time point.

Of the 11 transcripts studied, only two transcripts encoding a wheat orthologue of ornithine decarboxylase (ODC) and an isoform of arginine decarboxylase designated as ADC2 (see below) showed a significant increase following inoculation with *F. graminearum *(Figure 5). At one day post inoculation, ODC was induced seven fold compared to mock albeit with some variability (*P *= 0.054). Transcripts for *ODC *were significantly induced at two days post-inoculation (~40-fold controls, *P *= 0.003) and continued to increase up to three days post-inoculation where a level >100-fold that of mock controls was reached and maintained for the seven day duration of the experiment.

In the model plant *Arabidopsis*, two genes designated as *ADC1 *and *ADC2 *encode separate isoforms of ADC. *ADC1 *and *ADC2 *are functionally redundant as mutants for either gene are viable but double mutants are not [38]. Interestingly, *ADC2*, but not *ADC1*, has been shown to be responsive to salt and other abiotic stresses in Arabidopsis [39,40]. Similarly, we identified two distinct *ADC *sequences in wheat. However, based on nucleotide and amino acid comparisons between Arabidopsis and cereal ADC sequences, which one of these sequences is the direct wheat orthologue of the Arabidopsis *ADC2 *gene could not be established. Of these two sequences, only one was inducible during FHB disease development (Figure 5A and data not shown), suggesting that similarly to Arabidopsis, different *ADC *isoforms are differentially regulated under stress in wheat. We therefore designated the pathogen-inducible wheat ADC gene as *ADC2*. This gene was induced three-fold and ~10-fold, relative to mock-inoculated controls, at two and four days post-inoculation (Figure 5A).

To further characterize the relative speed of induction we compared the expression patterns of *ODC *and *ADC2 *to those of the peroxidase encoding gene *TaPERO*, which is known to be transcriptionally induced in wheat following inoculation by *F. graminearum, F. culmorum *or *F. pseudograminearum *[41,42]. *TaPERO *was induced similarly to *ODC *and *ADC2 *with three-fold induction observed at one day post-inoculation, followed by a rapid increase between two and three days post-inoculation relative to mock-inoculated controls (Figure 5C). This is consistent with *ODC *and *ADC2 *induction being part of the coordinated defence response to FHB.

### The polyamine pathway is co-ordinately regulated in the rachis under infection

**Figure 6:**
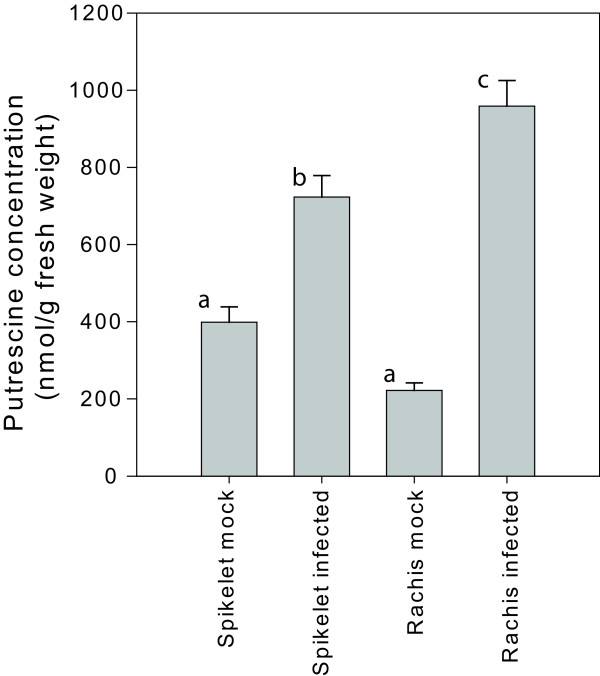
**Putrescine concentration in dissected wheat head material under Fusarium head blight infection**. n = 8 individual heads. Letters above bars represent statistically different groupings (Tukey test *P *< 0.05).

**Figure 7:**
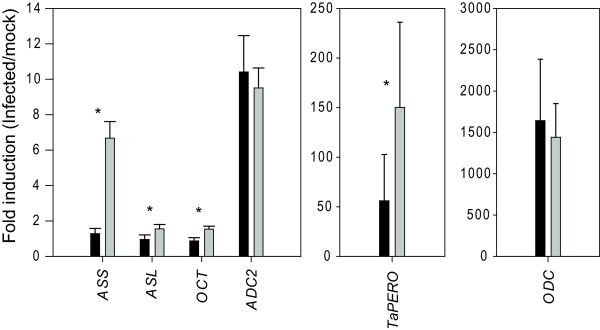
**Comparison of polyamine pathway gene expression in dissected head tissue under Fusarium head blight infection**. Values are relative to mock-treated heads. Black bars are spikelet material and grey bars are rachis material. Asterisks indicate statistically significant differences (P < 0.05) as described in the text. Error bars are the standard error of the mean, n = 8 individual heads. *ASS*, argininosuccinate synthase; *ASL*, argininosuccinate lyase; *OCT*, ornithine carbamoyl transferase; *ADC2*, arginine decarboxylase 2; *TaPERO*, peroxidase; *ODC*, ornithine decarboxylase.

It is now well known that one of the roles that DON plays during FHB development is to allow the fungus to colonise the rachis of infected spikelets and spread into the rachis of the spike [4,43]. Additionally, our previous work has demonstrated *TRI5 *is strongly expressed in the rachis tissue of wheat [18]. Given the importance of rachis in disease spread within the infected head, we were particularly interested in knowing whether the polyamine pathway is specifically or especially induced in this tissue during FHB development. To test this, we collected the rachis and the spikelets separately from spray- and mock-inoculated heads at six day post-inoculation. Putrescine quantification and quantitative RT-PCR was then carried out on each material. In mock-inoculated heads, putrescine levels were not significantly different in the rachis and spikelets (Figure 6). However, under infection, levels of putrescine in the rachis increased more dramatically than those in the spikelets (Figure 6; difference between infected rachis and spikelets *P *= 0.011, mock versus infected for both spikelets and rachis *P *< 0.001). Similar levels of induction of *ODC *and *ADC2 *were observed in both rachis and spikelets. However, transcripts encoding for argininosuccinate synthase (*P *= 2×10^-4^) and to a lesser extent argininosuccinate lyase (*P *= 0.04) and orthinine carbamoyl transferase (*P *= 0.001) all showed significantly higher levels of induction in rachis material (Figure 7). *ASS, ASC and OCT *showed no significant transcriptional induction in whole head samples. The enzymes encoded by these genes catalyse earlier steps in the polyamine biosynthetic pathway than ODC and ADC (Figure 1) suggesting a more coordinated induction of the pathway may occur in the rachis following infection.

### Polyamine induction is a general response of resistant and susceptible wheats to FHB

**Figure 8:**
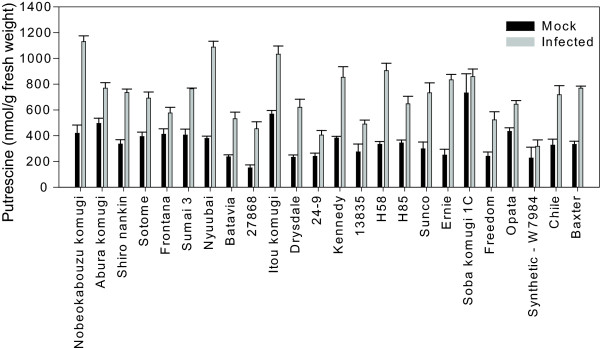
**Putrescine levels in mock and inoculated wheat heads of diverse wheat cultivars**. Measurements were taken at three days post inoculation. Error bars are the standard error of the mean n≥4. Genotypes are plotted in increasing levels of DON as described in the Materials and Methods.

To test whether activation of the polyamine biosynthetic pathway by *F. graminearum *correlates with resistance or susceptibility to FHB, we investigated the responses of 24 diverse bread wheat lines that have been reported to vary in their response to FHB [44]. As described in the Material and Methods, we first confirmed in independent inoculation experiments that these 24 lines were indeed different in their response in FHB disease symptom development and DON accumulation. Overall, there was a good correlation (r = 0.75) between DON levels and disease susceptibility across these lines, supporting the already known link between DON and disease symptom development in these lines. These lines were also inoculated and whole heads sampled at three days post-inoculation and analysed for polyamine and amino acids and the data for putrescine is shown in Figure 8. Considerable variation in the levels of putrescine, from 150 to 732 nmoles/g fresh weight in mock and from 318 to 1130 nmoles/g fresh weight in inoculated heads, was evident across the genotypes (Figure 8). As described earlier for cv. Kennedy (Figure 2), we found a significant induction of putrescine production in 22 of the wheat lines tested with the exception of Soba komugi 1C and Synthetic-W7984 (Figure 8). Similar inductions were observed for spermidine in all the lines examined (Additional File 2). Cadaverine detection (data not shown) and spermine levels (Additional File 2) were variable across the genotypes. No amino acid, including the potent DON inducer arginine showed any induction upon infection at three days post inoculation in these lines (data not shown). These results, therefore, indicate that the induction of putrescine and spermidine is a general response of wheat to infection and occurs in both resistant and susceptible lines.

We tested the correlations between putrescine and spermidine levels found in both mock and inoculated heads and FHB disease ratings and DON levels in these wheat lines (see Material and Methods) but no significant correlation was found (data not shown). These results suggest that polyamine induction is a general response of both FHB resistant and susceptible wheats to infection.

## Discussion

Several factors are known to influence the production of trichothecenes by *F. graminearum *in culture [13-15,18]. However, whether these factors also affect toxin production *in planta *during pathogenesis is unknown. The plant stress metabolites polyamines are one of the most potent toxin inducing factors in culture and result in toxin and biosynthetic enzyme mRNA levels equivalent to those observed in infected heads [18]. Here we have tested whether it is plausible that polyamines may act as *in planta *inducers of trichothecene production in wheat. By analysing the temporal changes in polyamine levels and the expression of the genes for their biosynthesis in relation to the biosynthesis of the trichothecene DON during FHB disease development. A key finding was that the induction of expression of key polyamine biosynthetic pathway genes and increase in polyamine (e.g. putrescine) levels following inoculation of wheat heads preceded the detection of DON in infected tissue. Induction of ODC by FHB was also observed in publically available global expression data in both resistant and susceptible near isogenic lines for Fhb1/fhb1 in wheat, and to a lesser extent during barley FHB [45-47]. Furthermore the timing of induction of *ADC2 *and *ODC *was similar to that of *TaPERO *known to be induced in response to *Fusarium *spp. [41,42]. These observations are consistent with a model where the early stages of fungal challenge of wheat flowers induce polyamine biosynthesis as a generic host stress response and this response is sensed by the pathogen as a cue for boosting trichothecene mycotoxin production such as DON.

It is well established that DON production in the fungus is not required for the initial infection of wheat flowers. However, DON is required as a virulence factor facilitating fungal colonisation and disease spread from the initially infected floret to other florets via the rachis [4,48]. In the absence of fungal DON production, the progress of *F. graminearum *is halted at the rachis node by plant cell wall thickenings as part of the normal defence response [4]. The model proposed above where the fungus initially stimulates a stress response in the host and then uses this as a trigger for DON production is also consistent with the proposed role for DON later in the infection process rather than it being necessary for initial infection processes.

The importance of the rachis node for induction of DON production *in planta *has recently been elegantly demonstrated [43]. The tight tissue specificity for DON production observed in the rachis tissue suggests that any DON-inducing factors are likely to be preferentially synthesised in this zone. Interestingly, Peeters et al. [35] detected only putrescine and no other polyamines in the rachis during wheat anthesis, suggesting some tissue specificity in the production of this particular polyamine that is also a potent inducer of DON production. In our analysis of transcripts for polyamine biosynthesis from whole heads we observed significant induction of only *ODC *and *ADC2 *but when the rachis was sampled separately there was also a significant induction of transcripts for three other enzymes earlier in the pathway suggesting a coordinated induction of the biosynthetic steps to putrescine. This is also consistent with the model proposed for putrescine as an inducer of DON production during infection.

The concentration of putrescine reached in heads following inoculation was approximately 0.5 mM based on a sample fresh weight basis. In culture, the lower concentration limit observed for the induction of DON by polyamine inducers such as putrescine is approximately 1 mM (data not shown) which is higher than the putrescine concentrations measured here *in planta*. Although this may argue against a possible in *planta *DON-inducing role for this metabolite, it should be noted that the actual putrescine concentration at the host-pathogen interface may be higher than the overall tissue level and it is possible that DON is synergistically induced by multiple compounds and conditions as demonstrated previously [15,18]. Also, it is possible that infection hyphae may differ in their sensitivity to inducing compounds when compared to vegetative hyphae growing in batch axenic culture conditions. More definitive information on the role of polyamines may be obtained using transgenic plants silenced for ODC and/or ADC2 that contain significantly reduced levels of polyamines in heads. However, given the central importance of polyamines in many biological processes and the branched biosynthesis pathway in wheat, plants with reduced polyamine levels maybe difficult to generate. Fungal mutants that are non-responsive to the polyamine signals may also be useful to definitively test this hypothesis.

Our analysis of infected wheat heads could not discriminate between polyamines and amino acids of fungal and plant origin. However, the early induction of polyamines such as putrescine, before fungal biomass increases appreciably together with the increases observed in transcripts of genes encoding plant polyamine biosynthetic enzymes all provide evidence that the wheat plant is the major contributor of the metabolic changes observed. Indeed PCR analysis of fungal polyamine biosynthetic genes showed all genes analysed were constantly expressed during infection (data not shown). One polyamine, cadaverine, was not detected in the non-inoculated heads and increased in concentration in parallel with fungal biomass. Cadaverine is produced by decarboxylation of lysine but no clear homologue of the bacterial lysine decarboxylase sequences were identified in the genome sequence of *F. graminearum *(data not shown). In plants it is highly likely that decarboxylation of lysine is a result of the ODC enzyme acting on lysine as an alternative substrate [49]. This may become significant when ODC transcripts, and presumably enzyme levels, were so strongly induced by infection. This would explain why a delay is observed in the production of cadaverine in infected tissue.

## Conclusions

In summary, the *in planta *induction of DON biosynthesis in *F. graminearum *is a complex process to which polyamines are likely to contribute. Future work to more specifically define the role of polyamines as inducers of DON during infection will require functional tests with fungal mutants with impaired perception of polyamines as well as tissue specific localisation of metabolites at the host-pathogen interface.

## Methods

### Plant material and inoculation

The susceptible Australian bread wheat (*Triticum aestivum *L.) cultivar Kennedy was used for all experiments unless indicated otherwise. Four seeds were sown in 10 cm pots in potting mix amended with osmocote, and grown in a controlled environment room with a photoperiod of 14 h, at 25°C and 50% relative humidity. The light intensity was 500 mmol m^-2 ^s^-1^. Night-time temperature was set at 15°C, with 90% relative humidity.

Unless specified otherwise, plants were spray inoculated at mid-anthesis with *F. graminearum *isolate CS3005 [50]. Approximately 2 mL of a 1×10^6 ^spores mL^-1 ^or water for mock inoculations was applied to each head using a Preval Sprayer (Precision Valve Corporation, NY, USA). Heads were covered with humidified plastic zip lock bags following treatment. Plastic bags were replaced three days post-inoculation with a glassine bag. All analyses were carried out on individual heads.

A panel of 24 bread wheat accessions that differ for resistance to FHB [44] were selected to screen for differential accumulation of polyamines and DON during FHB following spray inoculation. The response of these genotypes to FHB inoculation and their DON accumulation was assayed following point inoculation of heads as described previously [51], except disease scores and DON assays were conducted 14 days after infection. The genotypes selected and their respective DON accumulation (ppm in fresh head tissue) and disease scores (proportion of spikelets with symptoms) were as follows: Nobeokabouzu komugi (11.3 ppm, 0.127), Abura komugi (13.7, 0.147), Shiro nankin (14.2, 0.158), Sotome (15.7, 0.128), Frontana (16.2, 0.122), Sumai 3 (16.3, 0.173), Nyuubai (17.3, 0.144), Batavia (17.5, 0.171), 27868 (19.7, 0.125), Itou komugi (21.5, 0.165), Drysdale (24.5, 0.349), 24-9 (26.0, 0.145), Kennedy (26.4, 0.344), 13835 (26.5, 0.328), H58 (30.3, 0.123), H85 (31.0, 0.214), Sunco (32.4, 0.182), Ernie (33.1, 0.185), Soba komugi 1C (38.4, 0.370), Freedom (44.4, 0.177), Opata (50.3, 0.331), W7984 (50.9, 0.427), Chile (94.2, 0.537), and Baxter (100.6, 0.381).

### Polyamine and amino acid detection and quantification

Polyamines were quantified as previously described [18]. Free amino acids were extracted by grinding the heads under liquid nitrogen and resuspending a known quantity (up to 500 mg) of the powder in 2 mL of methanol, incubated at -20°C overnight and then 8 mL of water added and centrifuged to pellet solid matter. 500 μL of supernatant was dried down under vacuum. This extract was used with the Waters (Milford, MA, USA) AccQ-Tag amino acid detection kit, with quantification by HPLC according to the manufacturer's instructions. In a separate experiment, to allow more sensitive quantification of ornithine, the amino acid analysis was performed using the Waters AccQ-Tag Ultra chemistry at the Australian Proteome Analysis Facility (Sydney, Australia).

### Reverse transcriptase quantitative polymerase chain reaction

RT-qPCR was carried out as previously described [11]. To determine the target sequence of wheat polyamine genes for primer design, first the rice locus encoding the target gene was identified by a combination of text querying of putative function using the rice genome and reciprocal blast comparisons with the relevant Arabidopsis loci. The rice loci numbers were then used to query the Wheat Estimated Transcript Server (WhETS) database [52] to determine the orthologous wheat sequences. Where WhETS identified multiple homeologous sequences, ClustalW [53] alignments were used to determine conserved regions of these sequences for design of primers. Where suitable conserved regions could not be identified, multiple primer pairs were utilised. Primers were designed using the Primer3 software [54]. Primer sequences used to amplify wheat polyamine genes and the orthologous rice loci are listed in Table 1. Fungal biomass accumulation was estimated by comparing 18 S rRNA amplification from the fungus and plant respectively using primers listed in Table 1. *TRI5 *gene expression was measured relative to fungal 18 S using primers listed in Table 1.

### Deoxynivalenol quantification

Deoxynivalenol was quantified using the ELISA kit from Beacon Analytical Systems (Saco, Maine, USA) as per the manufacturer's instructions.

## Authors' contributions

DMG, KK, SP, FJT, JMM designed the research. DMG, SP and AR performed the experiments and analysed data. DMG, KK and JMM wrote the manuscript. All authors read and approved the manuscript.

## Supplementary Material

Additional file 1 **Free amino acids quantified during Fusarium head blight infection of wheat**. Free amino acids quantified during Fusarium head blight infection of wheat. Open circles denote infected samples, closed circles are mock inoculated. Error bars represent the standard error of the mean, n = 4. Click here for file

Additional file 2 **Spermidine and spermine concentrations in mock- and Fusarium head blight infected diverse wheat lines**. Spermidine (A) and spermine (B) concentrations in mock- and Fusarium head blight infected diverse wheat lines. Error bars are the standard error of the mean n≥4. Genotypes are plotted in increasing order of DON concentration. Click here for file
