# Supplementary figures and images for: Early activation of wheat polyamine biosynthesis during Fusarium head blight implicates putrescine as an inducer of trichothecene mycotoxin production
Source: BMC Plant Biol. 2010 Dec 30;10:289. doi: 10.1186/1471-2229-10-289 (PMC3022911; doi:10.1186/1471-2229-10-289)

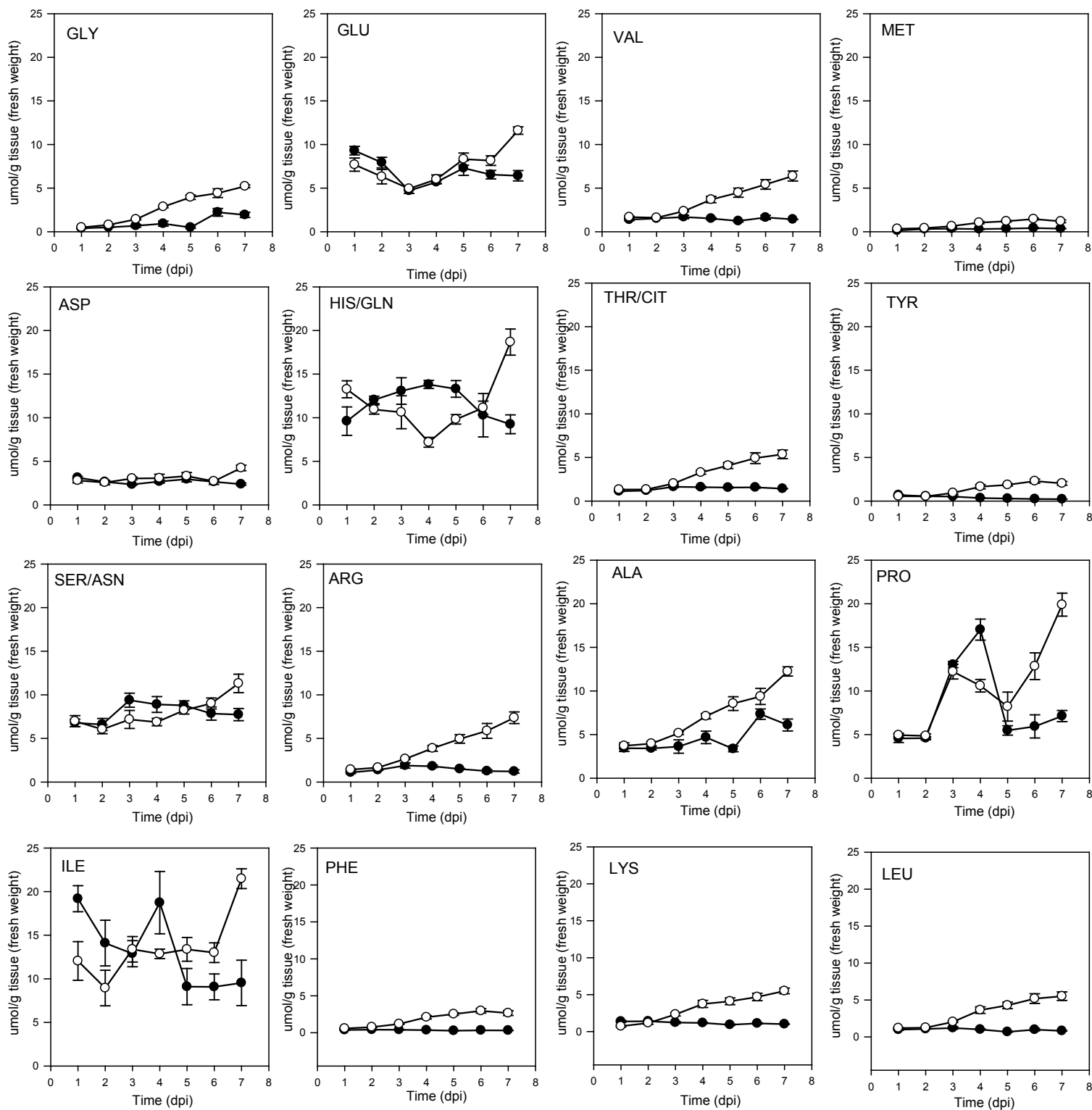

Supplement: Additional file 1 — Free amino acids quantified during Fusarium head blight infection of wheat. Free amino acids quantified during Fusarium head blight infection of wheat. Open circles denote infected samples, closed circles are mock inoculated. Error bars represent the standard error of the mean, n = 4. [file 1471-2229-10-289-S1.PDF]

**A**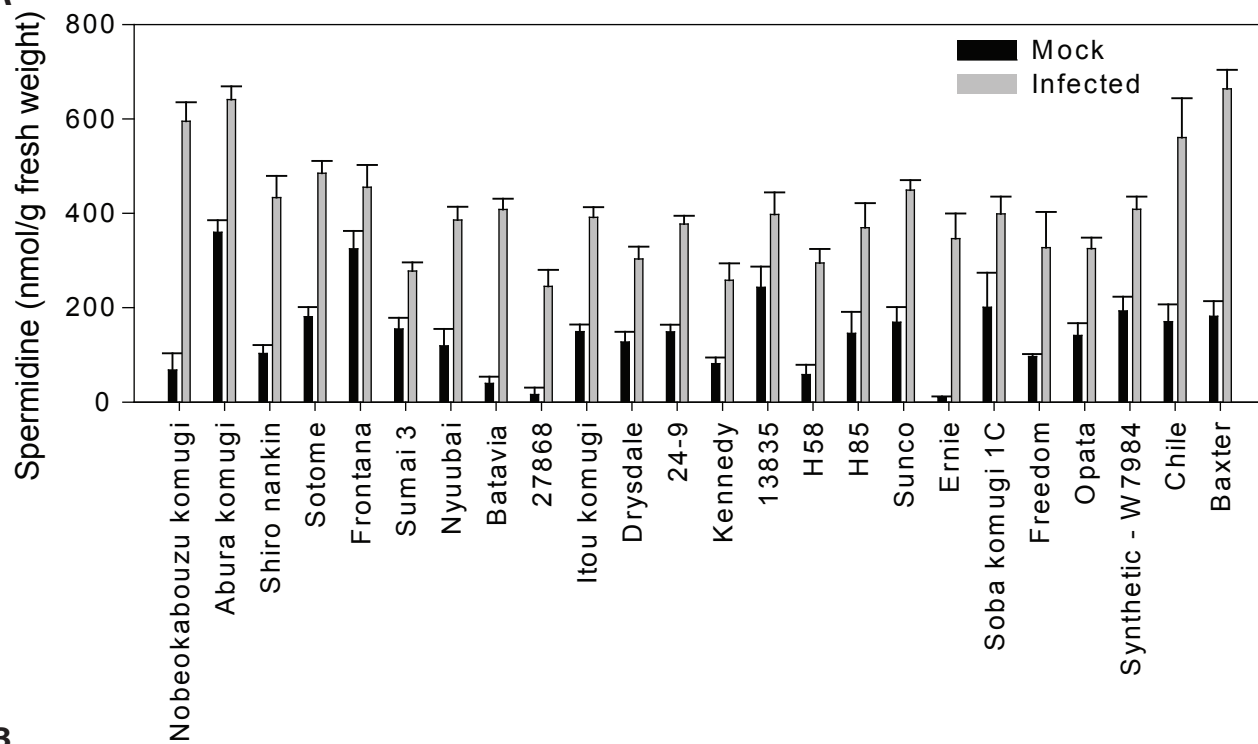**B**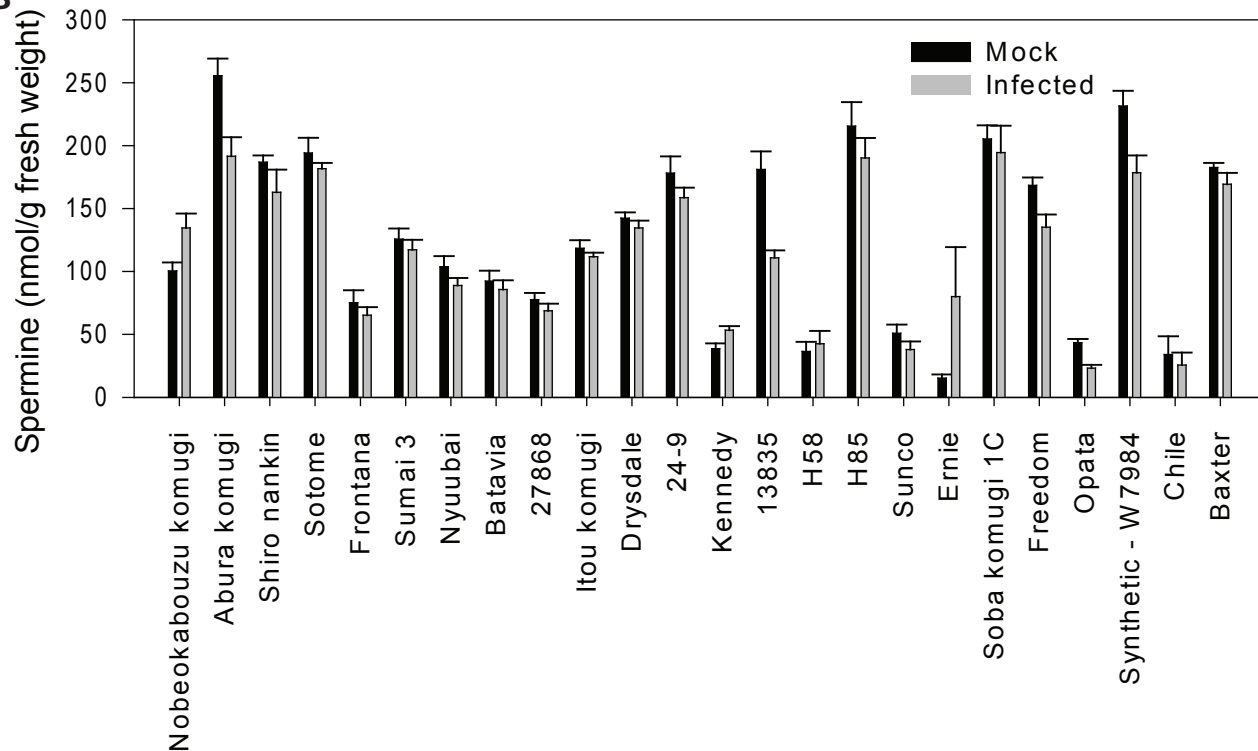

Supplement: Additional file 2 — Spermidine and spermine concentrations in mock- and Fusarium head blight infected diverse wheat lines. Spermidine (A) and spermine (B) concentrations in mock- and Fusarium head blight infected diverse wheat lines. Error bars are the standard error of the mean n≥4. Genotypes are plotted in increasing order of DON concentration. [file 1471-2229-10-289-S2.PDF]
